# USP36 SUMOylates Las1L and Promotes Its Function in Pre–Ribosomal RNA ITS2 Processing

**DOI:** 10.1158/2767-9764.CRC-24-0312

**Published:** 2024-10-30

**Authors:** Yanping Li, Yunhan Yang, Rosalie C. Sears, Mu-Shui Dai, Xiao-Xin Sun

**Affiliations:** Department of Molecular and Medical Genetics, School of Medicine, and the OHSU Knight Cancer Institute, Oregon Health & Science University, Portland, Oregon.

## Abstract

Ribosome biogenesis is a highly regulated cellular process requiring a large cohort of accessory factors to ensure the accurate production of ribosomes. Dysregulation of ribosome biogenesis is associated with the development of various human diseases, including cancer. The Las1L–Nol9 endonuclease–kinase complex is essential for the cleavage of the rRNA internal transcribed spacer 2 (ITS2), the phosphorylation of the 5′-hydroxyl end of the resulting precursor, and, thus, the maturation of the 60S ribosome. However, how the Las1L–Nol9 complex is regulated in cells is unclear. In this study, we report that the nucleolar ubiquitin-specific protease USP36 is a novel regulator of the Las1L–Nol9 complex. USP36 interacts with both Las1L and Nol9 and regulates their stability via deubiquitination. Intriguingly, USP36 also mediates the SUMOylation of Las1L, mainly at lysine (K) 565. Mutating K565 to arginine (R) does not affect the levels of Las1L and the formation of the Las1L–Nol9 complex, but abolishes its function in ITS2 processing, as unlike wild-type Las1L, the K565R mutant failed to rescue the defects in the ITS2 processing induced by the knockdown of endogenous Las1L. These results suggest that USP36-mediated Las1L SUMOylation is critical for ITS2 processing and that USP36 plays a critical role in ribosome biogenesis by regulating the Las1L–Nol9 complex.

**Significance:**

This study identifies USP36 as a deubiquitinating and small ubiquitin-like modifier ligase dual-function enzyme to mediate Las1L deubiquitination and SUMOylation. Las1L SUMOylation at K565 plays a critical role in pre-rRNA ITS2 processing. Thus, our study reveals a novel downstream pathway for USP36-regulated ribosome biogenesis.

## Introduction

Eukaryotic ribosome biogenesis is a multistep and highly orchestrated cellular process of making ribosomes. It starts with the transcription of 47S pre-rRNA by RNA polymerase I from rDNA genes in the nucleolus. Co-transcriptional assembly of ribosomal proteins and accessory factors into the nascent pre-rRNAs forms the 90S pre-ribosome particles, which undergo an endonucleolytic pre-rRNA cleavage to generate pre-40S and pre-60S ribosome particles (1–3). Both particles are then subjected to further maturation processes involving rRNA cleavage, modification, and folding in the nucleolus and nucleoplasm and are exported to the cytoplasm to form functional ribosomes (1–3). During the stepwise maturation of the pre-ribosome subunits, several hundreds of accessory factors, including proteins and small nucleolar RNAs, are critically involved (1–3). Among these factors, the Las1L–Nol9 endonuclease–kinase complex (Las1–Grc3 complex in yeast) is required for the rRNA internal transcribed spacer 2 (ITS2) processing (4–9). The endonuclease Las1L cleaves the 32S rRNA at the ITS2 (5, 6, 9). The resulting 12S rRNA product with 2′,3′-cyclic phosphate is further processed by the RNA exosome to generate 5.8S rRNA (10–12), whereas the 5′-hydroxyl end of the ITS2 is phosphorylated by Nol9 (7, 8, 13) to mark ITS2 degradation by the 5′ to 3′-exonuclease XRN2 (Rat1 in yeast; refs. 14–16) and the maturation of 28S rRNA. Thus, the Las1L–Nol9 endonuclease–kinase complex is essential for the 60S ribosomal subunit biogenesis. Nonetheless, how the Las1L–Nol9 complex is regulated during ribosome biogenesis is less understood.

Recent studies including our work have revealed that SUMOylation, a posttranslational modification of proteins by small ubiquitin-like modifiers (SUMO), plays a critical role in ribosome biogenesis (17, 18). A number of ribosome biogenesis accessory factors are regulated by SUMOylation, including Las1L (4, 19), Pelp1 (20), NPM (21, 22), nucleolin (23), and small nucleolar ribonucleoprotein (snoRNP) complex components Nop58, Nop56, Nhp2, and DKC1 (24–26). Proteomic studies also found that ribosome biogenesis–related proteins are one of the major groups of SUMOylated proteins (27–31). On the other hand, deSUMOylation is also important for ribosome biogenesis. DeSUMOylation of NPM by the SUMO-specific protease SENP3 is critical for 28S rRNA maturation and the subsequent nucleolar export of the 60S pre-ribosomal subunit (32). Transient SUMOylation of Pelp1 facilitates the recruitment of MDN1 to remodel pre-60S ribosome subunits and their translocation from the nucleolus to nucleoplasm, whereas the subsequent deSUMOylation of Pelp1 by SENP3 leads to the release of the MDN1–Pelp1 complex from pre-60S ribosome subunits and their recycling back to the nucleolus (20). Similarly, SUMOylation also promotes the translocation of Las1L from the nucleolus to nucleoplasm, and SENP3 depletion results in the accumulation of Las1L in the nucleoplasm (4, 19). However, how Las1L is SUMOylated in cells is not clear.

In this study, we identified the nucleolar ubiquitin-specific protease USP36 as a novel regulator for the Las1L–Nol9 endonuclease–kinase complex. We show that USP36 interacts with and deubiquitinates both Las1L and Nol9 and regulates their stability. Interestingly, USP36 also acts as a SUMO ligase to promote Las1L SUMOylation, mainly at K565. Wild-type (WT) Las1L, but not its SUMO-defective K565R mutant, rescued the defects of the ITS2 processing caused by the knockdown of endogenous Las1L. Thus, SUMOylation at K565 is critical for Las1L’s function in the ribosome biogenesis.

## Materials and Methods

### Cell culture and transfection

Human H1299, HeLa, and HEK293 cells were cultured in DMEM supplemented with 10% FBS, 50 U/mL penicillin, and 0.1 mg/mL streptomycin at 37°C in a 5% CO_2_ humidified atmosphere. These cell lines were obtained from ATCC. Cell lines were passaged fewer than 30 times for a maximum of 2 months and routinely monitored for *Mycoplasma* contamination. Manufacturers performed authentication through short tandem repeat profiling. Plasmid transfection was conducted using Lipofectamine 2000 (Life Technologies) for HEK293 cells and TransIT-LT1 reagents (Mirus Bio) for HeLa and H1299 cells following the manufacturers’ protocols.

### Plasmids, antibodies, and reagents

Flag-tagged USP36 and its deletion mutants as well as V5-tagged USP36 and its catalytically inactive C131A mutant were described previously (25, 33, 34). The Flag-tagged Las1L plasmid (pFlag-CMV-Las1L) was kindly provided by Dr. Catherine Denicourt (The University of Texas Health Science Center; ref. 4). Flag-Las1L deletion mutants were constructed by PCR cloning. The PCR products were digested with *Bgl*II and *Eco*RI and inserted into the pcDNA3-2Flag vector at *Bam*HI/*Eco*RI sites. Nol9 cDNA amplified from HeLa cells was inserted into the pcDNA3-2Flag vector at *Eco*RI/*Xba*I sites to generate the Flag-Nol9 plasmid. All Flag-tagged Nol9 deletion mutants were also constructed by inserting PCR products into the pcDNA3-2Flag vector at *Eco*RI/*Xba*I sites. The GFP-Nol9 plasmid was a gift from Dr. Robin E. Stanley (National Institute of Environmental Health Sciences, NIH; ref. 7). Flag-*Las1L*^K565R^, Flag-*Las1L*^K226R^, Flag-*Las1L*^K241R^, Flag-*Las1L*^K565R;K569R^, and Flag-*Las1L*^K565R;K241R^ mutants were generated by site-directed mutagenesis using the QuikChange Kit (Agilent Technologies). All primers for PCR cloning and site-directed mutagenesis are listed in Supplementary Table S1. His-SUMO1, His-SUMO2, and His-ubiquitin (Ub) plasmids were described previously (25, 35, 36). The Flag-Las1L cDNAs were also subcloned into the pcDNA4-TO vector (Life Technologies) to generate pcDNA4-TO-Flag-Las1L and pcDNA4-TO-Flag-Las1L^K565R^ plasmids. These plasmids were then used to construct the Las1L_siRNA_res plasmids by mutagenesis to generate siRNA-resistant Las1L (WT and the K565R mutant), in which the Las1L siRNA targeting the sequence 5′-CAC​CAA​GAC​TGG​ACG​GAA​T-3′ was mutated to 5′-TACGAAAACAGGTAGAAAC-3′.

Anti-Las1L (A304-438A, Bethyl Laboratories, RRID: AB_2620632), anti-Nol9 (16083-1-AP, Proteintech, RRID: AB_11124314), anti-USP36 (14783-1-AP, Proteintech, RRID: AB_2213357), anti-PELP1 (A300-180A, Bethyl Laboratories, RRID: AB_242526), anti-WDR18 (A15875, ABclonal, RRID: AB_2763304), anti-TEX10 (17372-1-AP, Proteintech, RRID: AB_2201871), anti-SENP3 (5591, Cell Signaling Technology, RRID: AB_10694546), anti-RPL30 (s-98106, Santa Cruz Biotechnology, RRID: AB_2181770), anti-Nop58 (A302-719A, Bethyl Laboratories, RRID: AB_10755121), anti-Flag (M2, F3165, Sigma, RRID: AB_259529), anti-V5 (R960-25, Life Technologies, RRID: AB_2556564), anti-SP1 (07-645, EMD Millipore), anti–digoxigenin-AP (11093274910, Roche), and anti-tubulin (66240-1-Ig, Proteintech, RRID: AB_2881629) were purchased. Rabbit anti-USP36 serum was provided by Dr. Masayuki Komada (Tokyo Institute of Technology, Japan; refs. 33, 37). Rabbit polyclonal anti-SUMO1 and anti-SUMO2/3 antibodies were provided by Dr Yoshiaki Azuma (University of Kansas). Anti-RPL5, anti-RPL11, and anti-RPS27a were generated as previously described (38–40). RNase A and RNase T1 (Thermo Fisher Scientific) were purchased.

### Immunoblot and co-immunoprecipitation analyses

Cells were lysed in lysis buffer consisting of 50 mmol/L Tris-HCl (pH 8.0), 0.5% Nonidet P-40, 1 mmol/L EDTA, 150 mmol/L NaCl, 1 mmol/L phenylmethylsulfonyl fluoride (PMSF), 1 mmol/L dithiothreitol, 1 μg/mL pepstatin A, and 1 mmol/L leupeptin with brief sonication. Equal amounts of protein were used for immunoblot (IB) analysis. Co-immunoprecipitation (Co-IP) was conducted by incubating equal amounts of cell lysates with anti-Flag (M2) antibody agarose gel (Sigma) at 4°C for 4 hours. The bound proteins were detected by IB analysis.

### RNAi

RNAi-mediated gene knockdown was performed essentially as previously described (34, 41). All the 21-nucleotide siRNA duplexes with a 3′-dTdT overhang were synthesized by Dharmacon, Inc. The target sequences are 5′-CAC​CAA​GAC​TGG​ACG​GAA​T-3′ (Las1L si-1, used for all experiments, except where indicated), 5′-CAG​AAA​CGC​AGA​AAG​CAC​A-3′ (Las1L si-2), 5′-GGC​CTT​CAG​TTA​ACT​GAG​A-3′ (Nol9), 5′-TGT​CCT​GAG​TGG​AGA​GAA​T-3′ (USP36 si-1, used for all experiments, except where indicated), 5′-GGA​AGA​GTC​TCC​AAG​GAA​A-3′ (USP36 si-2), and 5′-ACT​CCG​TAC​CAA​GGG​TTA​T-3′ (SENP3). The control scramble RNA was described previously (35). These siRNA duplexes (100 nmol/L) were introduced into cells using Lipofectamine 2000 (Invitrogen) following the manufacturer’s protocol. Cells were harvested 48 hours after transfection for IB.

### *In vivo* ubiquitination and SUMOylation assays

*In vivo* ubiquitination and SUMOylation assays under denaturing conditions were conducted using an Ni^2+^-NTA pulldown (PD) method as previously described (35, 36, 39). Briefly, the cells were transfected with His-Ub, His-SUMO1, or His-SUMO2 together with plasmids indicated in various experiments and treated with 20 μmol/L MG132 for 6 hours before harvesting. A total of 20% of the cells were used for direct IB, and the rest of the cells were subjected to Ni^2+^-NTA PD under denaturing conditions using lysis buffer consisting of 6 mol/L guanidinium-HCl; 0.1 mol/L Na_2_HPO_4_/NaH_2_PO_4_; 10 mmol/L Tris-HCl, pH 8.0; and 10 mmol/L β-mecaptoethanol. After washing, the bound proteins were eluted and analyzed using IB.

### Cell fractionation and Co-IP of nucleolar lysates

Nucleolar fractionation was performed as described previously (33). Briefly, the freshly harvested cells were washed with PBS, resuspended in hypotonic buffer A (10 mmol/L HEPES, pH7.8; 10 mmol/L KCl; 1.5 mmol/L MgCl_2_; and 0.5 mmol/L dithiothreitol) in the presence of PMSF, and incubated for 10 minutes on ice. The cells were homogenized using a B pestle douncer followed by spinning down at 3,000 rpm for 5 minutes at 4°C. The supernatant (cytoplasmic fraction) was then supplemented with one tenth volume of buffer B (0.3 mol/L Tris-HCl, pH 7.8; 1.4 mol/L KCl; and 30 mmol/L MgCl_2_). The nuclear pellets were washed with buffer A and then resuspended in buffer S1 (0.25 mol/L sucrose and 10 mmol/L MgCl_2_), layered over buffer S2 (0.35 mol/L sucrose and 0.5 mmol/L MgCl_2_), and centrifuged at 1,430 × *g* for 10 minutes at 4°C. The pelleted nuclei were resuspended in buffer S2 with PMSF and sonicated using a microtip probe at a power setting of 50%. The sonicated nuclei were then layered over buffer S3 containing 0.88 mol/L sucrose and 0.5 mmol/L MgCl_2_ and centrifuged at 3,000 × *g* for 10 minutes at 4°C. The pellet contained the purified nucleoli, and the supernatant represented the nucleoplasm. The nucleoli were then lysed in high-salt RIPA buffer containing 50 mmol/L Tris, pH 7.5; 500 mmol/L NaCl; 1% Nonidet P-40; 0.5% deoxycholate, and protease inhibitors in the presence of 80 U/mL DNase I on ice for 15 to 30 minutes. The lysates were then mixed with 2× volume of RIPA buffer without salt and left on ice for an additional 10 minutes, followed by centrifugation at a maximal speed for 15 minutes. The supernatant was then collected as a soluble nucleolar fraction for IP analysis (33).

### Immunofluorescence staining

Cells were fixed with 4% paraformaldehyde for 15 minutes with or without pretreating with 0.1% Triton X-100 for 1 minute. Then the cells were permeabilized with 0.25% Triton X-100 and blocked with 8% BSA. The cells were then stained with anti-Flag, anti-Las1L, or anti-SENP3 antibodies followed by Alexa Fluor 546 (red) goat anti-mouse antibody and Alexa Fluor 647 (far red) goat anti-rabbit antibody or Alexa Fluor 488 (green) goat anti-mouse antibody and Alexa Fluor 546 (red) goat anti-rabbit antibody (Life technologies) as well as 4′,6-diamidino-2-phenylindole to stain the DNA. The stained cells were analyzed under a Leica inverted fluorescence microscope.

### Northern blot

Nonradioactive Northern blot for rRNA processing was conducted as previously described (25, 34). Briefly, 4 μg of the total RNA was loaded onto agarose denaturing gels (6% formaldehyde/1.2% agarose in HEPES–EDTA buffer) and electrophoresed for 4 hours at 75 V. After washing, the gels were transferred to nylon membranes by capillarity overnight in 10× saline–sodium citrate. The membranes were UV cross-linked (120 mJ/cm^2^), followed by prehybridization in 50% formamide, 5× saline–sodium phosphate–EDTA, 5× Denhardt’s solution, 1% w/v SDS, and 200 μg/mL fish sperm DNA solution (Sigma) for 1 hour at 65°C. The digoxigenin-labeled oligonucleotide probe hybridizing to ITS2 (5′-CTG​CGA​GGG​AAC​CCC​CAG​CCG​CGC​A-3′) was added and incubated for 1 hour at 65°C and then overnight at 37°C. After washing with 2× saline–sodium citrate, the membranes were blocked in 1× blocking buffer (Roche) for 30 minutes at room temperature and incubated with anti-digoxigenin antibody (Roche, 1:10,000 dilution) for 30 minutes at room temperature, followed by washing twice, each 15 minutes, with washing buffer [0.1 mol/L maleic acid, 0.15 mol/L NaCl at pH 7.5, and 0.3% Tween 20 (v/v)]. After equilibration in detection buffer (0.1 mol/L Tris and 0.1 mol/L NaCl at pH 9.5), the membranes were incubated with chemiluminescent substrate CDP-Star (Roche, 1:200 dilution) at room temperature for 10 minutes and then exposed to films.

### Data availability

The data generated in this study are available within the article and its supplementary data file. Raw data generated in this study are available upon request from the corresponding author.

## Results

### USP36 interacts with the Las1L–Nol9 complex in the nucleolus

**Figure 1:**
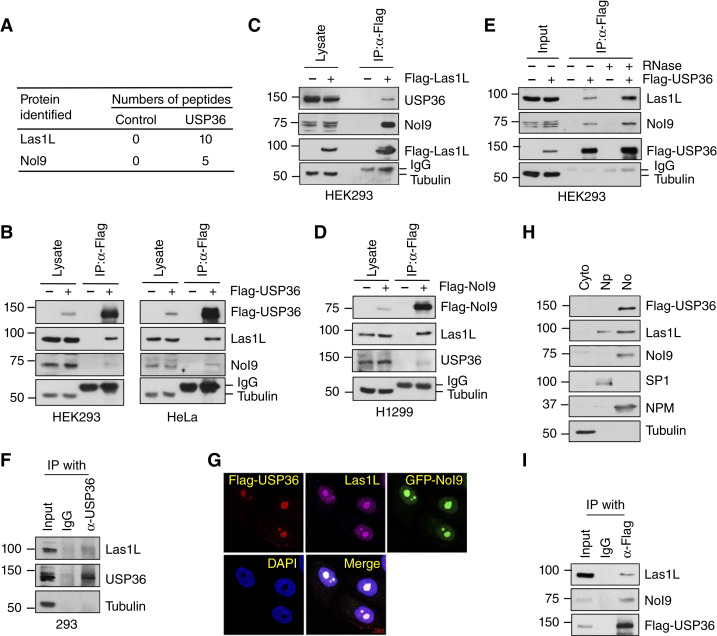
USP36 interacts with the Las1L–Nol9 complex. **A,** Number of Las1L and Nol9 peptides detected by mass spectrometry analysis of immunoprecipitates from control 293 and 293-Flag-USP36 cells by anti-Flag antibody. **B,** USP36 interacts with Las1L and Nol9. The HEK293 and HeLa cells transfected with Flag-USP36 were subjected to Co-IP with anti-Flag antibody, followed by IB. **C,** Co-IP of Las1L with USP36. The HEK293 cells transfected with Flag-Las1L were assayed by Co-IP using anti-Flag antibody, followed by IB. **D,** Co-IP of Nol9 with USP36. H1299 cells were transfected with Flag-Nol9 and assayed by Co-IP using anti-Flag antibody, followed by IB. **E,** The interaction of USP36 with Las1L–Nol9 is independent of RNA. The HEK293 cells transfected with Flag-USP36 were subjected to Co-IP with anti-Flag antibody in the presence or absence of 100 μg/mL RNase A and 100 U/mL RNase T1 treatment, followed by IB. **F,** Co-IP between endogenous USP36 and Las1L. 293 cell lysates were immunoprecipitated with anti-USP36, followed by IB with anti-Las1L antibody. **G,** Co-localization of Flag-USP36 with Las1L and Nol9. HeLa cells co-transfected with Flag-USP36 and GFP-Nol9 were immunostained with anti-Flag (Red) and anti-Las1L (far red), followed by 4′,6-diamidino-2-phenylindole (DAPI) staining for DNA (blue). **H,** Cell fractionation assays. HeLa cells stably expressing Flag-USP36 were fractionated into cytoplasmic (Cyto), nucleoplasmic (Np), and nucleolar fractions (No), followed by IB. Tubulin, SP1, and nucleophosmin (NPM) were used as cytoplasmic, nucleoplasmic, and nucleolar markers, respectively. **I,** Co-IP of USP36 with Las1L–Nol9 in the nucleolar fraction. Lysates from the nucleolar fraction shown in **H** were immunoprecipitated with anti-Flag antibody followed by IB.

USP36 has been shown to play a vital role in ribosome biogenesis (25, 34, 37, 42). To understand the underlying mechanism of USP36 regulation of ribosome biogenesis, we previously purified USP36-associated protein complexes (34) that contained both Las1L and Nol9 (Fig. 1A). The 60S ribosome biogenesis requires the cleavage of the 32S rRNA ITS2 by endonuclease Las1L and the phosphorylation of the 5′-hydroxyl group of the resulting precursor rRNA by Nol9 (4–9), which is essential for its further processing by XRN2 (14–16). Therefore, we sought to examine whether USP36 played a role in regulating the activity of the Las1L–Nol9 endonuclease–kinase complex. We first confirmed the interaction of Flag-USP36 with both Las1L and Nol9 in HEK293 and HeLa cells by Co-IP assays (Fig. 1B). Flag-Las1L (Fig. 1C) and Flag-Nol9 (Fig. 1D) also co-immunoprecipitated with USP36 in the cells. The interaction between USP36 and the Las1L–Nol9 complex was not abolished by the RNase treatment, indicating that this interaction is RNA independent (Fig. 1E). We also confirmed that endogenous USP36 interacts with endogenous Las1L (Fig. 1F). USP36 mainly localizes to the nucleolus (25, 33, 37, 43). Immunofluorescence staining showed that USP36 co-localizes with Las1L and Nol9 in the nucleolus (Fig. 1G). Cell fractionation assays also confirmed that the majority of the endogenous Las1L and Nol9 are present in the nucleolar fraction together with Flag-USP36 (Fig. 1H). Co-IP assay using the lysates from the isolated nucleolar fraction further confirmed that USP36 interacts with Las1L and Nol9 in the nucleolus (Fig. 1I). Together, these results suggest that USP36 interacts with the Las1L–Nol9 complex in the nucleolus.

### USP36 forms a complex with Las1L and Nol9

**Figure 2:**
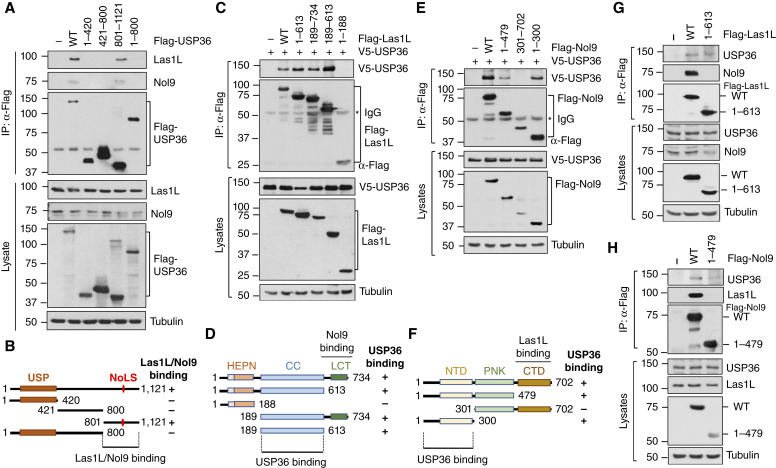
Mapping the interaction of USP36 with the Las1L–Nol9 complex. **A** and **B,** The C-terminal domain of USP36 binds to Las1L and Nol9. H1299 cells transfected with Flag-USP36 or its deletion mutants were assayed by Co-IP with anti-Flag antibody, followed by IB (**A**). Diagram of USP36 indicating the C-terminal Las1L–Nol9–binding domain is shown in **B**. USP, ubiquitin-specific protease. **C** and **D**, USP36 binds to the CC domain of Las1L. H1299 cells transfected with Flag-Las1L or its deletion mutants together with V5-USP36 were subjected to Co-IP with anti-Flag antibody, followed by IB (**C**). The diagram of Las1L domains is shown in **D**. HEPN, higher eukaryotes and prokaryotes nucleotide-binding domain; LCT, Las1L C-terminal tail; CC, coiled-coil. **E** and **F**, USP36 binds to the NTD domain of Nol9. H1299 cells transfected with Flag-Nol9 or its deletion mutants together with V5-USP36 were subjected to Co-IP with anti-Flag antibody, followed by IB (**E**). The diagram of Nol9 domains is shown in **F**. NTD, N-terminal domain; PNK, polynucleotide kinase; CTD, C-terminal domain. **G** and **H,** USP36 binds to Las1L and Nol9 independently. H1299 cells transfected with WT Flag-Las1L or the Nol9-binding defective mutant (**G**) or Flag-Nol9 or its Las1L-binding defective mutant (**H**) were subjected to Co-IP with anti-Flag antibody, followed by IB.

To characterize the interaction between USP36 and the Las1L–Nol9 complex, we sought to map which regions of USP36 bind to Las1L and Nol9. Using Co-IP–IB assays, we showed that both Las1L and Nol9 interact with the full-length and C-terminal nucleolar localization signal (NoLS)–containing regions, but not the N-terminal ubiquitin-specific protease domain and the central region, of USP36 (Fig. 2A). Thus, the Las1L–Nol9 complex binds to the C-terminus of USP36 (amino acids 801–1121; Fig. 2B), which is localized into the nucleolus (Supplementary Fig. S1) as previously reported (43). We also mapped where USP36 binds at Las1L and Nol9. Cells co-transfected with V5-USP36 and Flag-Las1L (Fig. 2C and D) or Flag-Nol9 (Fig. 2E and F) and their different deletion mutants were analyzed by Co-IP. As summarized in Fig. 2D and F, USP36 interacts with the middle coiled-coil domain of Las1L (amino acids 189–613) and the N-terminal domain (amino acids 1–300) of Nol9. Las1L and Nol9 form a higher-order complex containing at least two copies of Las1L and two copies of Nol9 (7, 13). Nol9 binds to the C-terminal tail domain of Las1L (amino acids 613–734) via its C-terminal domain (amino acids 479–702; ref. 7). To examine whether USP36 can interact with Las1L and Nol9 independently, we took advantage of the Flag-*Las1L*^1–613^ mutant, which lacks the Nol9-binding domain and thus does not bind to Nol9 and the Flag-*Nol9*^1–479^ mutant, which lacks the Las1L-binding domain and does not bind to Las1L. As shown in Fig. 2G and H, both mutants still interact with USP36. These results suggest that USP36 may form a complex with the Las1L–Nol9 complex by interacting with both proteins.

### USP36 deubiquitinates Las1L and Nol9 and regulates their levels

**Figure 3:**
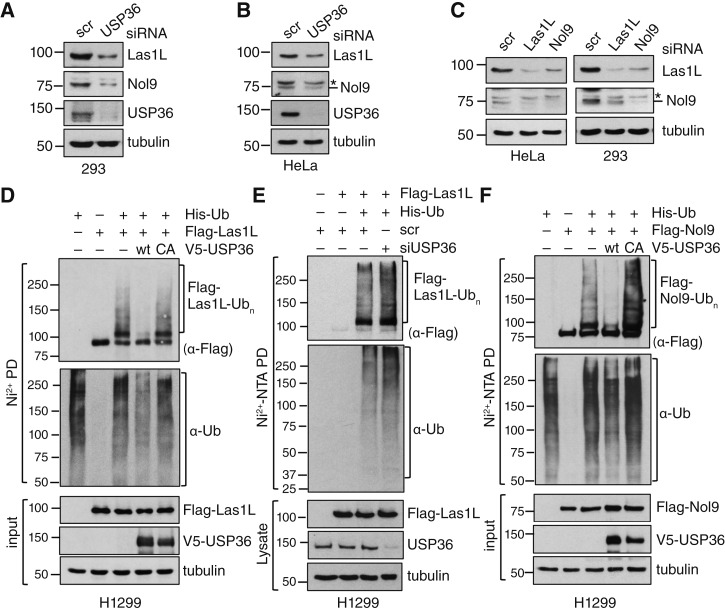
USP36 deubiquitinates Las1L and Nol9 and regulates their levels. **A** and **B,** Knockdown of USP36 reduces the levels of Las1L and Nol9. The HEK293 (**A**) and HeLa (**B**) cells transfected with scrambled (scr) or USP36 siRNA were assayed by IB. **C,** The stability of Las1L and Nol9 is dependent on each other. HeLa and HEK293 cells transfected with scr, Las1L, or Nol9 siRNA were assayed by IB. **D,** USP36 deubiquitinates Las1L. H1299 cells transfected with the indicated plasmids were subjected to Ni^2+^-NTA PD under denaturing conditions, followed by IB to detect the ubiquitinated species of Las1L. The protein expression is shown in the bottom panels. **E,** Knockdown of endogenous USP36 increases the ubiquitinated species of Las1L. H1299 cells transfected with the indicated plasmids together with scr or USP36 siRNA were subjected to Ni^2+^-NTA PD under denaturing conditions, followed by IB to detect the ubiquitinated species of Las1L. The protein expression is shown in the bottom panels. **F,** USP36 deubiquitinates Nol9. H1299 cells transfected with the indicated plasmids were subjected to Ni^2+^-NTA PD under denaturing conditions, followed by IB to detect the ubiquitinated species of Nol9. The protein expression is shown in the bottom panels.

Given that USP36 is a deubiquitinating (DUB) enzyme, we asked whether it deubiquitinates Las1L and Nol9 and regulates their levels. We observed that the knockdown of USP36 by siRNA markedly reduced the levels of both Las1L and Nol9 in HEK293 (Fig. 3A) and HeLa (Fig. 3B) cells. Similar to a previous observation that the stability of Las1 and Grc3 in budding yeast *Saccharomyces cerevisiae* depends on each other (5), we also observed that the knockdown of Las1L reduced Nol9 levels and vice versa in both HEK293 and HeLa cells (Fig. 3C). We then examined whether USP36 deubiquitinates Las1L and Nol9. *In vivo* ubiquitination assays using the Ni^2+^-NTA beads His purification method showed that overexpression of WT USP36, but not the catalytically inactive C131A mutant, deubiquitinates Las1L (Fig. 3D). Consistently, the knockdown of endogenous USP36 by siRNA increased the ubiquitinated species of Las1L (Fig. 3E). Also, overexpression of WT USP36, but not the C131A mutant, deubiquitinates Nol9 (Fig. 3F). Thus, USP36 acts as a DUB enzyme for the Las1L–Nol9 complex and is critical for maintaining their proper levels in cells.

### USP36 promotes Las1L SUMOylation

**Figure 4:**
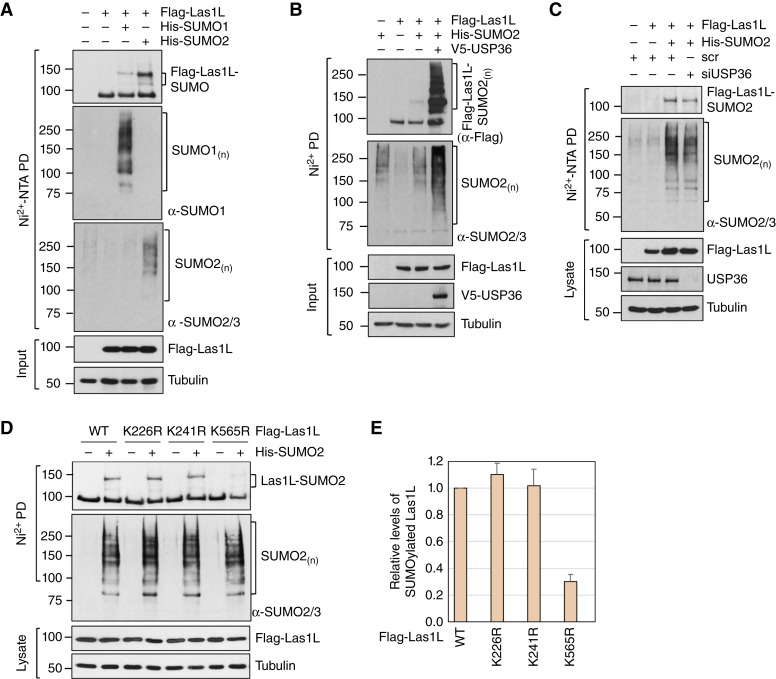
USP36 SUMOylates Las1L. **A,** Las1L is SUMOylated in cells. H1299 cells transfected with Flag-Las1L in the absence or presence of His-SUMO1 or His-SUMO2 were subjected to Ni^2+^-NTA PD under denaturing conditions, followed by IB with anti-Flag, anti-SUMO1, and anti-SUMO2/3 antibodies. **B,** USP36 promotes Las1L SUMOylation. H1299 cells transfected with the indicated plasmids were subjected to Ni^2+^-NTA PD, followed by IB to detect Las1L SUMOylation. The SUMOylated Las1L and total SUMOylated proteins are indicated. The protein expression is shown in the bottom panels. **C,** Knockdown of endogenous USP36 reduces Las1L SUMOylation. H1299 cells transfected with the indicated plasmids together with scr or USP36 siRNA were subjected to Ni^2+^-NTA PD, followed by IB to detect Las1L SUMOylation. The protein expression is shown in the bottom panels. **D** and **E,** Las1L is SUMOylated at K565. H1299 cells transfected with WT Las1L or the indicated lysine mutant plasmids were subjected to Ni^2+^-NTA beads PD under denaturing conditions, followed by IB. The SUMOylated Las1L and total SUMOylated proteins are indicated. The protein expression is shown in the bottom panels. The relative ratio of SUMOylated Las1L to native Las1L normalized to WT Las1L from the four individual experiments are shown in **E**.

We previously discovered that USP36 also functions as a SUMO ligase to promote SUMOylation of nucleolar proteins. Las1L has been shown to be SUMOylated in cells (4, 19). We therefore examined whether USP36 promotes Las1L SUMOylation. As shown in Fig. 4A, Las1L can be SUMOylated by both SUMO1 and SUMO2, albeit SUMO2 modification is stronger. Thus, we will focus on SUMO2 in this study. Co-expression of USP36 markedly increased the SUMOylated species of Las1L (Fig. 4B) and the knockdown of endogenous USP36 reduced the SUMOylation of Las1L (Fig. 4C), suggesting that USP36 acts as a SUMO ligase to SUMOylate Las1L. Several putative SUMOylation sites of Las1L, including two consensus Lys residues (K565 and K241) and two putative nonconsensus Lys residues (K226 and K569), are predicted using GPS-SUMO (44). Using Las1L deletion mutants, we indeed observed that SUMOylation occurs at the central coiled-coil domain of Las1L (amino acids 189–613) but not at its N-terminus (1–188; Supplementary Fig. S2A). To identify the SUMO acceptor lysine(s) in Las1L, we mutated individual Lys residues and performed *in vivo* SUMOylation assays under denaturing conditions. As shown in Fig. 4D and summarized in Fig. 4E, mutating K565, but not K241 and K226, to arginine (R) markedly abolished Las1L SUMOylation. Mutating K569 did not further reduce the SUMOylation of the *Las1L*^K565R^ mutant (Supplementary Fig. S2B). Thus, K565 is the major acceptor residue for Las1L SUMOylation.

### SUMOylation regulates Las1L nucleolar export and its association with pre-60S ribosome

**Figure 5:**
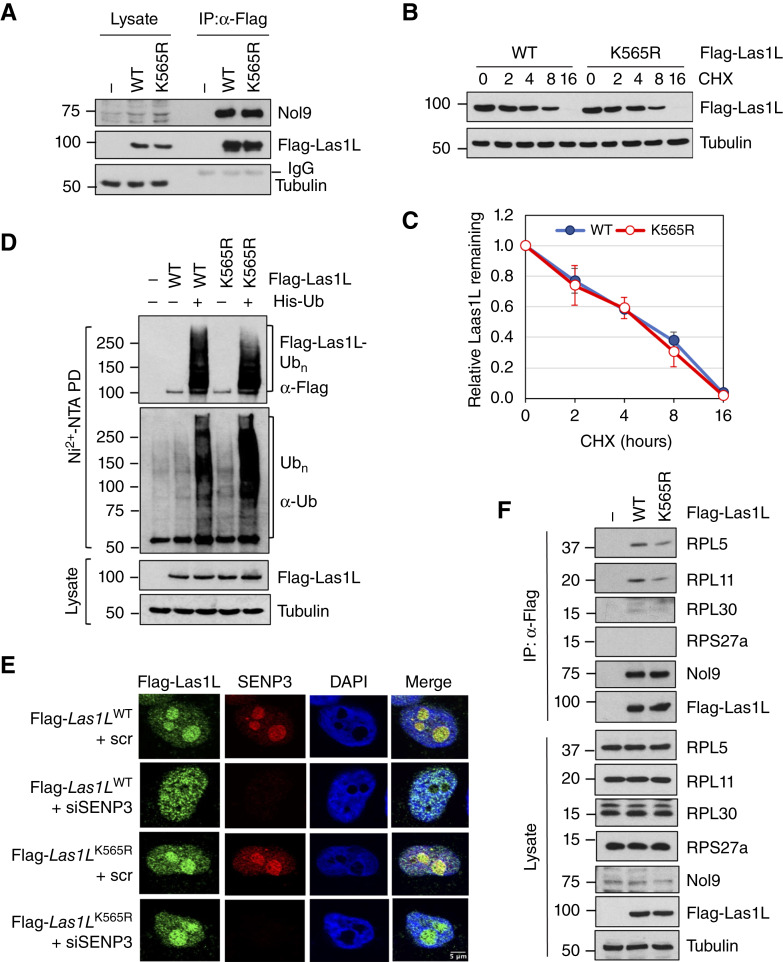
SUMOylation of Las1L regulates its cellular localization and association with the pre-60S ribosome. **A,** Las1L SUMOylation does not affect its interaction with Nol9. The HEK293 cells transfected with WT Las1L, the K565R mutant, or control vector were assayed by Co-IP with anti-Flag antibody, followed by IB. **B** and **C,** Mutating K565 does not affect the half-life of Las1L. HeLa cells transfected with WT Las1L or the K565R mutant were treated with 100 μg/mL cycloheximide (CHX) and harvested at different time points, followed by IB. A representative experiment is shown in **B**, and the quantification from the three independent experiments is shown in **C**. **D,** Mutating K565 does not affect Las1L ubiquitination determined by Ni^2+^-NTA beads PD under denaturing conditions. The ubiquitinated Las1L and total protein ubiquitination are indicated. The protein expression is shown in the bottom panels. **E,** Las1L SUMOylation at K565 affects its nucleoplasmic translocation. HeLa cells transfected with Flag-Las1L (WT or the K565R mutant) together with scr or SENP3 siRNA were immunostained with anti-Flag (green) and anti-SENP3 (red), followed by 4′,6-diamidino-2-phenylindole (DAPI) staining for DNA (blue). **F,** Mutating K565 attenuates the interaction of Las1L with the pre-60S ribosome. The HEK293 cells transfected with WT Flag-Las1L, the K565R mutant, or control vector were assayed by Co-IP with anti-Flag antibody, followed by IB to detect the indicated ribosomal proteins.

To understand the function of Las1L SUMOylation at K565, we first tested whether abolishing Las1L SUMOylation could affect the level of Las1L and the formation of the Las1L–Nol9 complex. As shown in Fig. 5A, mutating K565 does not affect the levels of Las1L or its interaction with Nol9. The half-life of the *Las1L*^K565R^ mutant is comparable with that of WT Las1L (Fig. 5B and C). Furthermore, mutating K565 did not significantly alter the ubiquitination of Las1L (Fig. 5D). Thus, Las1L SUMOylation at K565 does not affect Las1L ubiquitination and levels. Las1L SUMOylation has been shown to promote the translocation of Las1L to the nucleoplasm (4, 19). Consistently, we observed that the knockdown of SENP3 redistributes WT Las1L, but not the *Las1L*^K565R^ mutant, into the nucleoplasm (Fig. 5E; Supplementary Fig. S3). Our results further support that SUMOylation regulates the nucleolar export of Las1L. Las1L and Nol9 are components of the rixosome, a multiprotein complex critical for the remodeling and maturation of the pre-60S ribosome (4, 19, 20, 45, 46). To test whether USP36 interacts with the rixosome complex, we performed Co-IP experiments. Indeed, USP36 interacts with the rixosome components Pelp1, TEX10, WDR18, and SENP3 (Supplementary Fig. S4), suggesting that USP36 interacts with the rixosome to regulate 60S ribosome biogenesis. We previously showed that USP36 associates with the pre-60S ribosome (34). Thus, we next examined whether USP36 regulates Las1L association with the pre-60S ribosome. As shown in Fig. 5F, mutating K565 reduced the interaction of Las1L with the 60S ribosome subunits RPL5, RPL11, and RPL30, but not RPS27a—a subunit from the 40S ribosome that does not interact with Las1L. Together, these results suggest that USP36-mediated SUMOylation plays an important role in regulating the Las1L association with pre-60S ribosome and export.

### Las1L SUMOylation at K565 is critical for its function in ITS2 processing

**Figure 6:**
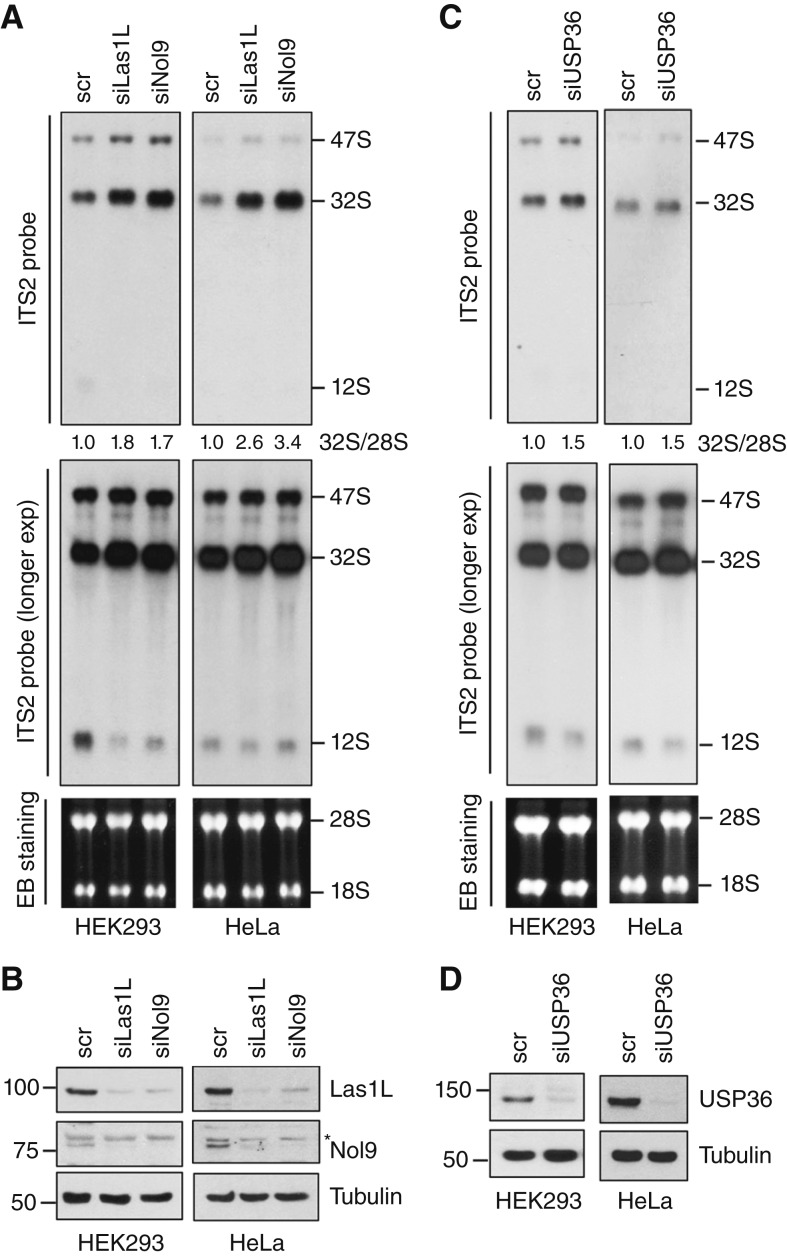
The Las1L–Nol9 complex and USP36 play a role in rRNA ITS2 processing. The HEK293 and HeLa cells transfected with scr, Las1L, Nol9 siRNA (**A** and **B**), or USP36 siRNA (**C** and **D**) were assayed for rRNA processing by Northern blot analysis using probe hybridizing to the ITS2 (**A** and **C**). The 47S pre-rRNA, 32S rRNA, 12S rRNA, and the relative ratios of 32S to 28S rRNA normalized to the scr control are shown in the top panels. Ethidium bromide (EB) staining of the RNA gels to indicate 28S and 18S rRNAs is shown in the bottom panels (**A** and **C**). The protein expression is shown by IB (**B** and **D**).

The Las1L–Nol9 endonuclease–kinase complex is essential for the processing of rRNA ITS2. Using Northern blot analysis with a probe hybridizing to ITS2, we confirmed that the knockdown of either Las1L or Nol9 by siRNAs markedly impaired ITS2 rRNA processing, leading to the accumulation of 47S and 32S precursors and reduction of 12S rRNA precursors in both HEK293 and HeLa cells (Fig. 6A and B). Consistent with its role in regulating Las1L, the knockdown of USP36 also leads to the accumulation of 47S and 32S precursors as well as the reduction of 12S rRNA products (Fig. 6C and D). These effects are less likely off-target effects, as the knockdown of Las1L or USP36 using different siRNAs also led to the accumulation of 47S and 32S precursors (Supplementary Fig. S5A and S5B).

**Figure 7:**
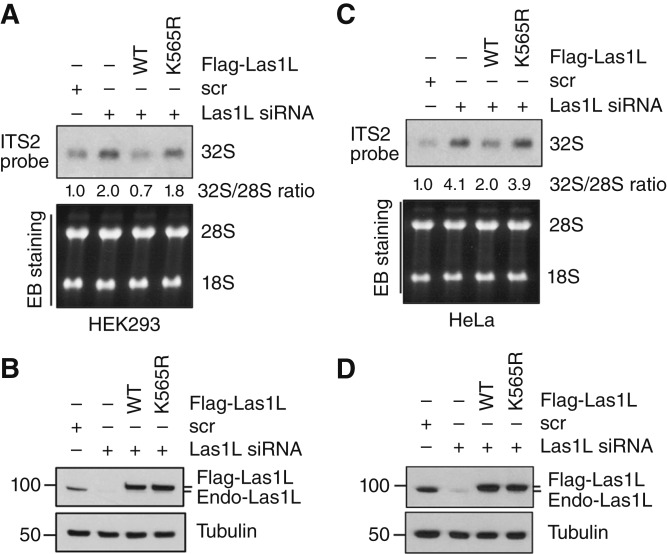
Las1L SUMOylation at K565 is critical for its function in rRNA ITS2 processing. The HEK293 (**A** and **B**) and HeLa (**C** and **D**) cells transfected with scr or Las1L siRNA together with siRNA-resistant WT Flag-Las1L or its K565R mutant were assayed for rRNA ITS2 processing by Northern blot analysis using the ITS2 probe. The 32S rRNA precursor and the relative ratios of 32S to 28S rRNA normalized to the scr control are indicated in the top panels. Ethidium bromide (EB) staining of the RNA agarose gel is shown in the bottom panels (**A** and **C**). One representative experiment from three independent experiments is shown. The expression of the endogenous Las1L and exogenous Flag-Las1L is shown by IB (**B** and **D**).

To further understand the role of Las1L SUMOylation in ribosome biogenesis, we performed the Las1L knockdown and rescue experiments. Although re-expression of siRNA-resistant WT Las1L largely rescued the 32S rRNA accumulation caused by the knockdown of endogenous Las1L, the siRNA-resistant *Las1L*^K565R^ mutant failed to do so in both HEK293 (Fig. 7A and B) and HeLa (Fig. 7C and D) cells. Therefore, our results suggest that SUMOylation of Las1L by USP36 promotes Las1L function in pre-rRNA ITS2 processing and thus ribosome biogenesis.

## Discussion

USP36 was initially identified as a nucleolar DUB enzyme implicated in nucleolar structure and function in ribosomal biogenesis by deubiquitinating and stabilizing several ribosome biogenesis factors, such as B23 and fibrillarin (37, 43); the RNA helicase DHX33 (42); and the largest subunit of RNA Pol I Rpa190 (47). USP36 also deubiquitinates and stabilizes *c*-Myc (33, 48), a master regulator of ribosome biogenesis promoting transcription by all three classes of RNA polymerases (47), thus coordinating ribosome biogenesis with cell-cycle progression. Intriguingly, we recently revealed that USP36 also acts as a SUMO ligase promoting the SUMOylation of several nucleolar proteins, including the snoRNP components Nop58, Nop56, Nhp2, and DKC1 (25); the RNA exosome component EXOSC10 (34); and the microprocessor complex component DGCR8 (41). Thus, USP36 is emerging as a key ribosome biogenesis regulator.

In this study, we identified USP36 as a novel regulator of the Las1L–Nol9 endonuclease–kinase complex. USP36 interacts with both Las1L and Nol9 independent of RNA and acts as a SUMO ligase to promote Las1L SUMOylation. We further showed that Las1L SUMOylation at K565 is critical for its function in processing rRNA ITS2, as WT Las1L, but not the *Las1L*^K565R^ mutant, rescued the ITS2 processing defects in cells with knockdown of endogenous Las1L. Las1L lacks an NoLS region, and its nucleolar localization requires interaction with Nol9, which contains an NoLS region in its N-terminus (7). Las1L SUMOylation has been shown to promote its nucleoplasmic translocation (4, 19). We also observed that increasing Las1L SUMOylation by knocking down SENP3 results in the nucleoplasmic localization of Las1L, whereas the K565R mutant remains in the nucleolus upon SENP3 deletion. Thus, it is likely that the altered cellular localization of the K565R mutant would interfere with its function in processing ITS2. Further supporting this notion is the fact that the interaction of Las1L with the 60S pre-ribosome is attenuated by the K565R mutation (Fig. 5E). It remains to be tested whether SUMOylation could cause Las1L conformational changes, leading to increased endonuclease activity of Las1L. Consistent with the role of USP36 as the Las1L SUMO ligase, USP36 depletion also impairs the processing of ITS2, leading to the accumulation of 32S rRNA and downregulation of 12S rRNA, although USP36 has more diverse effects on rRNA processing by regulating other accessory factors such as the regulation of snoRNP protein group SUMOylation and RNA exosome (25, 34). Nevertheless, our finding reveals a novel mechanism of USP36 in ribosome biogenesis by regulating Las1L-mediated rRNA ITS2 processing.

Of note, USP36 also deubiquitinates both Las1L and Nol9 and regulates their levels. The knockdown of USP36 markedly decreased the levels of both Las1L and Nol9 proteins. This is in contrast with the regulation of other nucleolar substrates by USP36, such as snoRNP proteins (25), EXOSC10 (34), and DGCR8 (41), in which USP36 acts only as a SUMO ligase, but not as the DUB enzyme, and thus does not regulate their protein stability. As the K565 SUMOylation does not affect Las1L ubiquitination and turnover, our data suggest that USP36 regulates Las1L via both deubiquitination and SUMOylation, albeit K565 SUMOylation does not cross talk with Las1L ubiquitination.

Las1L has been shown to interact with the human Rix1 complex (Pelp1, Tex10, and WDR18), MDN1, and SENP3 to form the rixosome, a multiprotein complex critical for the remodeling and maturation of the pre-60S ribosomal subunits (4, 19, 20, 45). We showed that USP36 interacts with the rixosome components and that abrogating Las1L SUMOylation by the K565R mutation attenuated Las1L’s interaction with the pre-60S ribosome, suggesting that USP36 may regulate Las1L SUMOylation and 60S ribosome biogenesis in the context of the rixosome. Interestingly, several other rixosome components can also be SUMOylated. For example, SUMOylation of Pelp1 recruits the AAA ATPase MDN1 to remodel pre-60S ribosome subunits and their translocation from the nucleolus to the nucleoplasm, whereas deSUMOylation of Pelp1 by SENP3 leads to the release of the MDN1–Pelp1 complex from pre-60S ribosome subunits and their recycling back to the nucleolus (20). A recent study also showed that TEX10 can be modified by SUMO (49, 50), suggesting that the rixosome complex is likely subjected to regulation by group SUMOylation, similar to other SUMOylated protein complexes (25, 51, 52). It would also be interesting to test whether USP36 also promotes group SUMOylation of the rixosome complex and synergistically regulates their function in pre-60S ribosome maturation.

As USP36 is aberrantly overexpressed in various human cancers and is critical for ribosome biogenesis and cell growth (18, 53), it may be a promising therapeutic target in cancer. Notably, USP36 possesses DUB enzymatic and SUMO ligase dual activities that positively regulate cell growth and proliferation. Future work should aim to identify novel USP36 inhibitors targeting its DUB and SUMO ligase dual-enzyme activities for cancer treatment.

## Supplementary Material

Supplementary Figure S1 Supplementary Figure S1 shows the cellular localization of USP36 and its deletion mutants by IF.

Supplementary Table 1 Supplementary Table 1 lists all the primers for cloning and mutagenesis.

Supplementary Figure S2 Supplementary Figure S2 shows the SUMOylation of full-length Las1L and its N-terminal deletion mutant (aa 189–613) and that K565 is the main SUMOylation site.

Supplementary Figure S3 Supplementary Figure S3 Shows that Las1L SUMOylation at K565 affects its nucleoplasmic translocation.

Supplementary Figure S4 Supplementary Figure S4 shows that USP36 interacts with rixosome components including Pelp1, TEXT10, WDR18, and SENP3.

Supplementary Figure S5 Supplementary Figure S5 shows that siRNA-mediated knockdown of either Las1L or USP36 impairs the pre-rRNA ITS2 processing.
