# Supplementary material for: USP36 SUMOylates Las1L and Promotes Its Function in Pre–Ribosomal RNA ITS2 Processing
Source: Cancer Res Commun. 2024 Oct 30;4(10):2835–45. doi: 10.1158/2767-9764.CRC-24-0312 (PMC11523043; doi:10.1158/2767-9764.CRC-24-0312)
Supplement: Supplementary Figure S1 — shows the cellular localization of USP36 and its deletion mutants by IF. [file crc-24-0312_supplementary_figure_s1_suppsf1.pdf]

Supplementary Figure S1

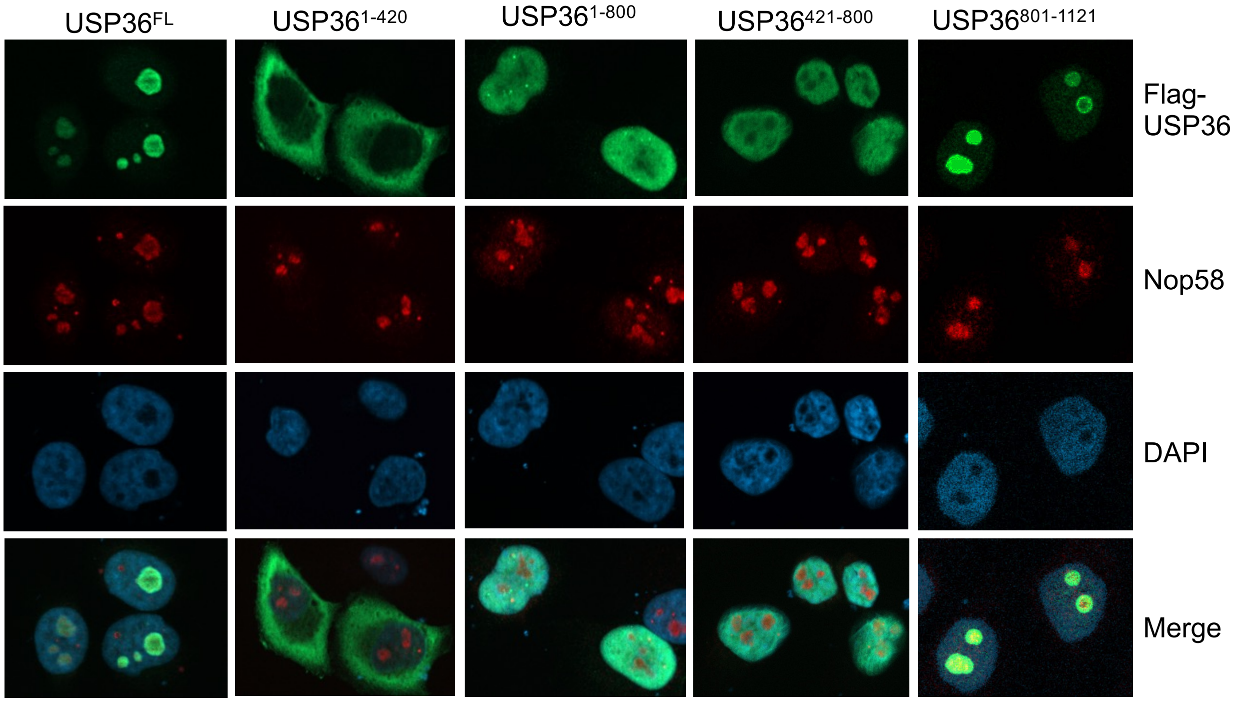

Supplementary Figure S1. **Cellular localization of USP36 and its deletion mutants determined by immunofluorescence staining.** H1299 cells transfected with Flag-tagged full-length (FL) USP36 and its deletion mutants were stained with anti-Flag and anti-Nop58 (a nucleolar marker) antibodies followed by DAPI staining for DNA.
