# Supplementary material for: USP36 SUMOylates Las1L and Promotes Its Function in Pre–Ribosomal RNA ITS2 Processing
Source: Cancer Res Commun. 2024 Oct 30;4(10):2835–45. doi: 10.1158/2767-9764.CRC-24-0312 (PMC11523043; doi:10.1158/2767-9764.CRC-24-0312)
Supplement: Supplementary Table 1 — lists all the primers for cloning and mutagenesis. [file crc-24-0312_supplementary_table_1_suppst1.pdf]

Supplementary Table 1: Primers for cloning and mutagenesis

Name	Sequence
Las1L_Bgl II_For	CGGAGATCTTCGTGGGAATCCGGGGCCGGGCCAG
Las1L_EcoRI_Rev	CCGGAATTCTCAGAAGAGCTGCAGGCCAG
Las1L 613 EcoRI-Rev	CCGGAATTCTCATGTAGAGAAAGGCCCCACCTCC
Las1L 189 Bgl II-For	CGGAGATCTGAGAACAGCCTGA GAGAGACC
Las1L 188 EcoRI_Rev	CCGGAATTCTCACAGTTGGCGGCACCAATAGG
Nol9_EcoRI_For	CGGGAATTCTGGCGGACTCGGGACTGCTGCTAAAG
Nol9_XbaI_Rev	CGGTCTAGATCACTTCATTTTTCGACAGAAC
Nol9 479 XbaI-Rev	CGGTCTAGATCATTCCAAAGCATCTGCAAATGC
Nol9 301 EcoRI-For	CCGGAATTCATGTCATTCTAGTTTGTGGATCC
Nol9 300 XbaI-Rev	CGGTCTAGATCAAGGGCAGCCATCTACTTCTTC
Las1L K565R-For	AGTGTTAATGATGTCAGGGAAGAGGAGAAGGAG
Las1L K565R-Rev	CTCCTTCTCCTCTTCCCTGACATCATTAACACTG
Las1L K226R-For	GACATCACAGAACAGAGACCAGAGCCTCAGGATG
Las1L K226R-Rev	ATCCTGAGGCTCTGGTCTCTGTTCTGTGATGTC
Las1L_K241R-For	ACGGAGTCAGATGTAAGGGCCGATGGAGACAGC
Las1L_K241R-Rev	GCTGTCTCCATCGGCCCTTACATCTGACTCCGT
Las1L_K565R_K569R-For	TGTCAGGGAAGAGGAGAGGGAGGAGAAAGAGGTC
Las1L_K565R_K569R-Rev	GACCTCTTTCTCCTCCCTCTCCTCTTCCCTGACA
