# Supplementary material for: USP36 SUMOylates Las1L and Promotes Its Function in Pre–Ribosomal RNA ITS2 Processing
Source: Cancer Res Commun. 2024 Oct 30;4(10):2835–45. doi: 10.1158/2767-9764.CRC-24-0312 (PMC11523043; doi:10.1158/2767-9764.CRC-24-0312)
Supplement: Supplementary Figure S2 — shows the SUMOylation of full-length Las1L and its N-terminal deletion mutant (aa 189–613) and that K565 is the main SUMOylation site. [file crc-24-0312_supplementary_figure_s2_suppsf2.pdf]

Supplementary Figure S2

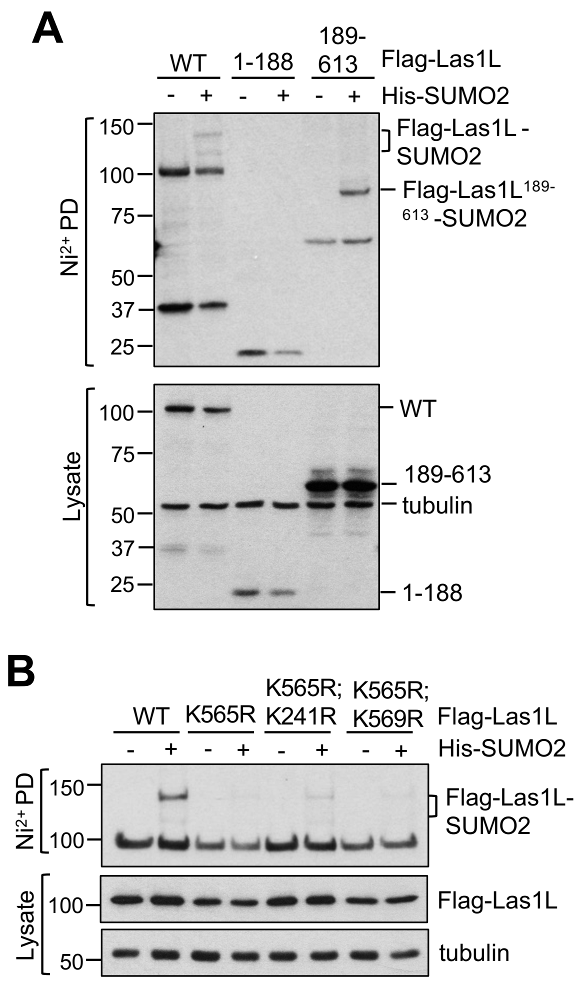

Supplementary Figure S2. **SUMOylation of Las1L. (A)** SUMOylation of Las1L deletion mutants. H1299 cells transfected with WT Las1L or its deletion mutants in the absence or presence of His-SUMO2 were subjected to Ni^{2+}-NTA PD followed by IB to detect Las1L SUMOylation. The SUMOylated WT Las1L and its fragment are indicated. The protein expression is shown at the bottom panel. **(B)**. Las1L is SUMOylated at K565. H1299 cells transfected with the indicated plasmids were subjected to Ni^{2+}-NTA PD followed by IB to detect Las1L SUMOylation. The SUMOylated Las1L is indicated. The protein expression is shown at the bottom panels.
