# Supplementary material for: USP36 SUMOylates Las1L and Promotes Its Function in Pre–Ribosomal RNA ITS2 Processing
Source: Cancer Res Commun. 2024 Oct 30;4(10):2835–45. doi: 10.1158/2767-9764.CRC-24-0312 (PMC11523043; doi:10.1158/2767-9764.CRC-24-0312)
Supplement: Supplementary Figure S3 — Shows that Las1L SUMOylation at K565 affects its nucleoplasmic translocation. [file crc-24-0312_supplementary_figure_s3_suppsf3.pdf]

Supplementary Figure S3

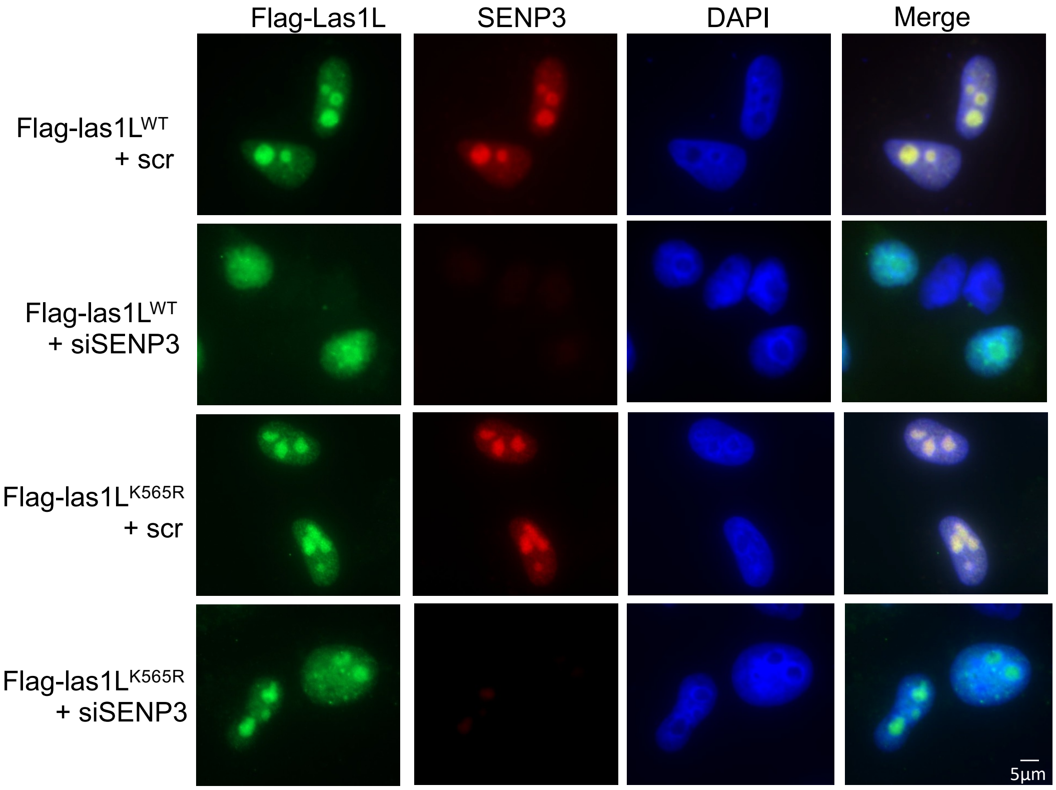

Supplementary Figure S3. **Las1L SUMOylation at K565 affects its nucleoplasmic translocation**. HeLa cells transfected with Flag-Las1L (WT or the K565R mutant) together with scr or SENP3 siRNA were immunostained with anti-Flag (Green) and anti-SENP3 (Red) followed by DAPI staining for DNA (blue).
