# Supplementary material for: USP36 SUMOylates Las1L and Promotes Its Function in Pre–Ribosomal RNA ITS2 Processing
Source: Cancer Res Commun. 2024 Oct 30;4(10):2835–45. doi: 10.1158/2767-9764.CRC-24-0312 (PMC11523043; doi:10.1158/2767-9764.CRC-24-0312)
Supplement: Supplementary Figure S4 — shows that USP36 interacts with rixosome components including Pelp1, TEXT10, WDR18, and SENP3. [file crc-24-0312_supplementary_figure_s4_suppsf4.pdf]

Supplementary Figure S4

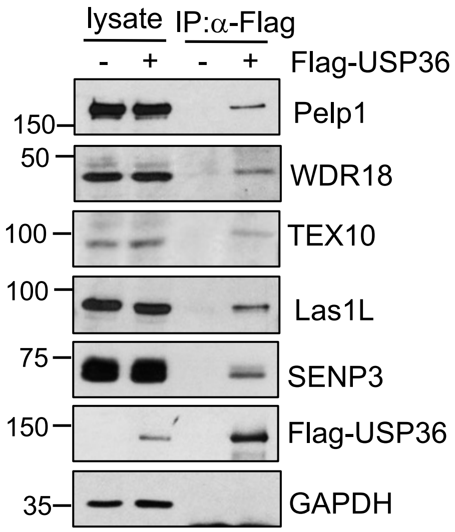

Supplementary Figure S4. **USP36 interacts with rixosome components.** 293 cells transfected with Flag-USP36 or empty vector were immunoprecipitated with anti-Flag antibody followed by IB detection of Pelp1, WDR18, TEX10, Las1L and SENP3.
