# Supplementary material for: USP36 SUMOylates Las1L and Promotes Its Function in Pre–Ribosomal RNA ITS2 Processing
Source: Cancer Res Commun. 2024 Oct 30;4(10):2835–45. doi: 10.1158/2767-9764.CRC-24-0312 (PMC11523043; doi:10.1158/2767-9764.CRC-24-0312)
Supplement: Supplementary Figure S5 — shows that siRNA-mediated knockdown of either Las1L or USP36 impairs the pre-rRNA ITS2 processing. [file crc-24-0312_supplementary_figure_s5_suppsf5.pdf]

Supplementary Figure S5

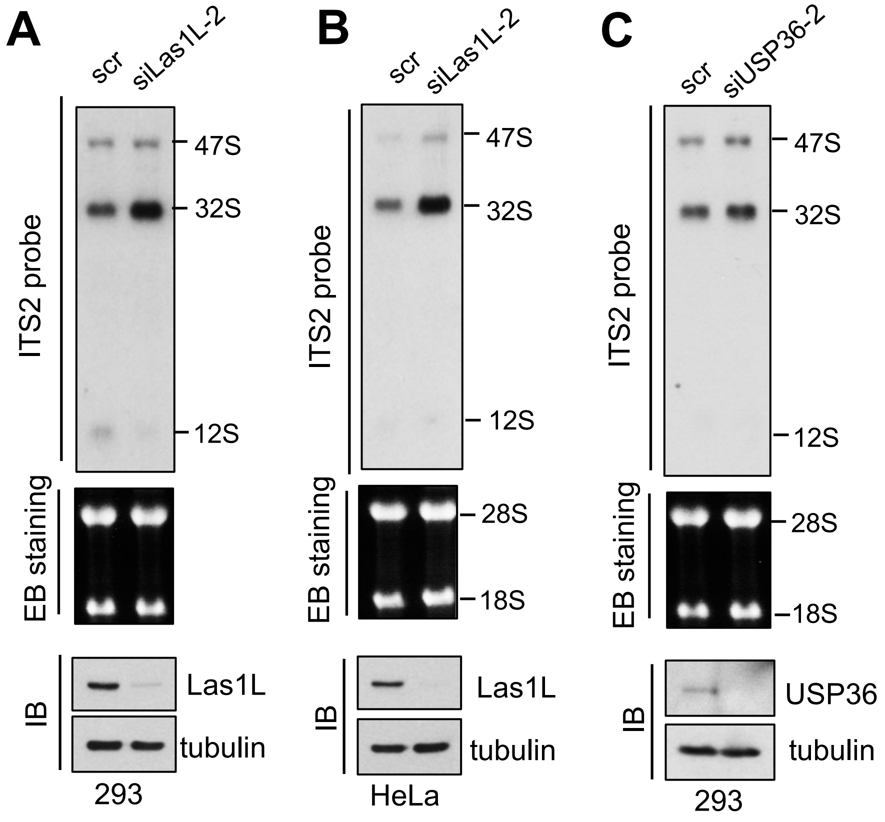

Figure S5. **Knockdown of Las1L or USP36 attenuates rRNA ITS2 processing.** 293 (A) and HeLa (B) cells transfected with scr or Las1L siRNA-2 and 293 cells transfected with scr or USP36 siRNA-2 (C) were assayed for rRNA ITS2 processing by Northern blot using ITS2 probe. The 32S rRNA precursor and the 12S rRNA product were shown in top panels. EB staining of the RNA agarose gel is shown in the middle panels. The expression of endogenous Las1L (A, B) and endogenous USP36 (C) is shown in the bottom panels.
